# Artificial Intelligence in Orthodontic Smart Application for Treatment Coaching and Its Impact on Clinical Performance of Patients Monitored with AI-TeleHealth System

**DOI:** 10.3390/healthcare9121695

**Published:** 2021-12-07

**Authors:** Andrej Thurzo, Veronika Kurilová, Ivan Varga

**Affiliations:** 1 Department of Stomatology and Maxillofacial Surgery, Faculty of Medicine, Comenius University in Bratislava, 81250 Bratislava, Slovakia; 2 Faculty of Electrical Engineering and Information Technology, Slovak University of Technology, 81219 Bratislava, Slovakia; veronika.hanuskova@gmail.com; 3 Institute of Histology and Embryology, Faculty of Medicine, Comenius University in Bratislava, 81372 Bratislava, Slovakia; ivan.varga@fmed.uniba.sk

**Keywords:** orthodontic treatment, clear aligners, smart application, AI, computerized learning, behavior change techniques, decision tree algorithm, telemedicine

## Abstract

Background: Treatment of malocclusion with clear removable appliances like Invisalign^®^ or Spark™, require considerable higher level of patient compliance when compared to conventional fixed braces. The clinical outcomes and treatment efficiency strongly depend on the patient’s discipline. Smart treatment coaching applications, like strojCHECK^®^ are efficient for improving patient compliance. Purpose: To evaluate the impact of computerized personalized decision algorithms responding to observed and anticipated patient behavior implemented as an update of an existing clinical orthodontic application (app). Materials and Methods: Variables such as (1) patient app interaction, (2) patient app discipline and (3) clinical aligner tracking evaluated by artificial intelligence system (AI) system—Dental monitoring^®^ were observed on the set of 86 patients. Two 60-day periods were evaluated; before and after the app was updated with decision tree processes. Results: All variables showed significant improvement after the update except for the manifestation of clinical non-tracking in men, evaluated by artificial intelligence from video scans. Conclusions: Implementation of application update including computerized decision processes can significantly enhance clinical performance of existing health care applications and improve patients’ compliance. Using the algorithm with decision tree architecture could create a baseline for further machine learning optimization.

## 1. Introduction

Prevalence of malocclusion and orthodontic treatment need is well researched. Noticeable incisor irregularity occurs in the majority of all ethnic groups, with only 35% of adults having well-aligned mandibular incisors [1]. Irregularity is severe enough in 15% that both social acceptability and function could be affected, and major arch expansion or extraction of some teeth would be required for correction. About 20% of the population have deviations from the ideal bite relationship; in 2% these are severe enough to be disfiguring and are at the limit for orthodontic correction. Application of the Index of Treatment Need to the survey data reveals that 57% to 59% of each racial/ethnic group has at least some degree of orthodontic treatment need [1].

Both clear aligners and braces are effective in malocclusion treatment. Clear aligners had advantage in segmented movement of teeth and shortened treatment duration, but were not as effective as braces in producing adequate occlusal contacts, controlling teeth torque, and retention [2]. 

Clear Aligners therapy (CAT) is considered efficient orthodontic treatment. It aligns and levels the arches; and is effective in controlling anterior intrusion but not anterior extrusion; it is effective in controlling posterior buccolingual inclination but not anterior buccolingual inclination; it is effective in controlling upper molar bodily movements; and is less effective in controlling rotation of rounded teeth in particular [3]. 

However, CAT is currently under intense innovative technological pressure including new optimized attachment systems with high potential to use whole exposed tooth surface for application of forces to achieve proper tooth movements. This article is focused on support of modern CAT with smart mobile application and effect of its artificial intelligence (AI) upgrade.

### 1.1. Mobile Applicationsin Orthodontics

Mobile applications (apps) are to be a crucial tool in management of modern aesthetical and comfortable treatments where patient compliance is the key. They already play an increasingly important role in daily life and patients’ social networks like Instagram represents an aid to the standard verbal motivation performed by orthodontists towards young patients under an orthodontic treatment [4]. With the number of orthodontic-related apps continuing to increase, and the rapid development of artificial intelligence, the potential to yield tremendous benefits to both clinicians and patients is apparent. More advanced features of artificial intelligence have been introduced to orthodontic applications recently. For example, three-dimensional convolutional neural networks (3D CNN) have high potential for automatized 3D cephalometric evaluation directly from the Cone-Beam Computed Tomography (CBCT) or facial growth predictions [5].

These advanced forms of artificial intelligence can overtake also the process of orthodontic auxiliaries designing where currently dominates Finite Element Analysis (FEM) [6].

If orthodontic apps are to become mainstream and obtain greater acceptance, scientific validation and investigation of these apps are to be undertaken. The current situation in the clinical field shows only 20 publications about apps used in orthodontics. Their structure is expanded in the Table 1. In summary:

- 8 studies were Randomized Controlled Trials (RCTs) (35%)
- 10 were case-controls (53%)
- 1 was a cohort study (retrospective) (6%)
- 1 cross-sectional study was found (6%)

Seven studies (35%) were based on apps used for diagnostics, and all were cephalometric apps. 7 studies (41%) investigating apps used for reminders were present. 4 studies (24%) investigated dedicated remote monitoring apps and all four studied Dental Monitoring [7,8].

In comparison to app analyzed in this research paper (StrojCHECK^®^, Bratislava, Slovakia, 3Dent Medical, osim.sk (accessed on 1 November 2021)—Society for Medical Innovation (SMI)), most of the current apps used for orthodontic purposes, are simple apps without back-end or any Artificial Intelligence (AI) implementations. There is no publication about orthodontic apps other than simple reminders, basic diagnostics (cephalometry) or remote monitoring. Most of the app regarding orthodontic therapy are focused on oral hygiene status and coaching [12,29]. Despite the current weak scientific coverage, there are no doubts tele-orthodontics is the future of dental digitalization [30,31,32,33]. 

With the current situation described above, it is worth to highlight that over 90% of all apps used in orthodontics are single apps without any server back-end as well as they possess any truly intelligent behavior. Potential of implementations of machine learning algorithms and other levels of artificial intelligence features might bring significant leap in their clinical efficiency. This paper describes effect of an update of existing orthodontic app with AI algorithms of decision processes. 

Technologies in our mobiles transformed almost every aspect of our lives. Smartphones enable patients to request, receive, and transmit information irrespective of the time and place. Also, the global pandemic has forced healthcare providers to employ TeleHealth technology to help handling this tense situation [34].

### 1.2. Tele-Orthodontics—Dental Monitoring and Other Aspects of TeleHealth

Tele-orthodontics—Dental Monitoring^®^ (DM) (Dental Monitoring Co., Paris, France) with distant monitoring is current reality in orthodontics. We can, as the clinical orthodontists proactively monitor our patients with virtual examinations to supplement chairside appointments. Though this approach is tainted with negative connotations associated with the direct to patient business model [27,30], there are undisputable advantages of remote monitoring to the clinical practice of orthodontics [35].

Interesting aspect of this research paper is a true clinical evaluation of the impact of the app AI update. Performance of the app before and after the update was different. To evaluate clinical impact an AI Tele-Health system was used (DM). This system is capable to evaluate various clinical situations from the patients’ home-made video-scans [26]. AI evaluation recognizes various clinical situations like: loss of attachment, loss of various accessories, gingivitis, caries and many other. In this paper this telemedicine system is used for frequent clinical evaluation of aligners tracking on the teeth. This AI TeleHealth system is frequently used in other studies and its accuracy and reliability is well evaluated [23,26,27,36,37,38]. How this study examined the true clinical performance of patients with this system is described in Material and methods chapter in more detail.

Dental Monitoring is reducing (not eliminating) the need for in-office visits. However, nearly half of the studies currently published on this topic comparing clinical treatment with DM and without DM, frequently misunderstand the focus of this technology. The spotlight of this AI-powered DM is not on reduction of patient’s visits rather enhanced level of control over treatment development. As described earlier in this paper, the CAT in general, is prone to patients’ indiscipline. Patient not wearing aligners properly (more than 22 h per day), results in situation called “non-tracking”. This manifests as a discrepancy between shape of the aligner and real teeth position. This can be evaluated with DM. Possible lower frequency of physical visits to dental clinic is only secondary. However, it is beneficial for both the orthodontist and the patient, as the orthodontist can improve treatment and chairside efficiency, while the patients can avoid the extra financial and time costs of traveling to the practice [35,38]. The key point remains that DM setup protocol allows better control over the treatment despite they might result even in more frequent patient visits as every lost attachment is noticed and the alarms are triggered. In contrary the frequency of non-DM-patient checkups are defined by the orthodontist and loss of attachment might be overlooked and despite longer time between in-office visits of such a patient, the treatment with CAT with missing attachments will probably result in aligner non-tracking or even a necessity to restart the treatment.

Dental monitoring protocols are not paradigm shifting to older orthodontic techniques like fixed vestibular orthodontics treatments. These require a frequent chairside activation. On the other hand, customized appliances such as CAT may take full advantage of DM due to the preprogrammed tooth movement [38].

**Figure 1:**
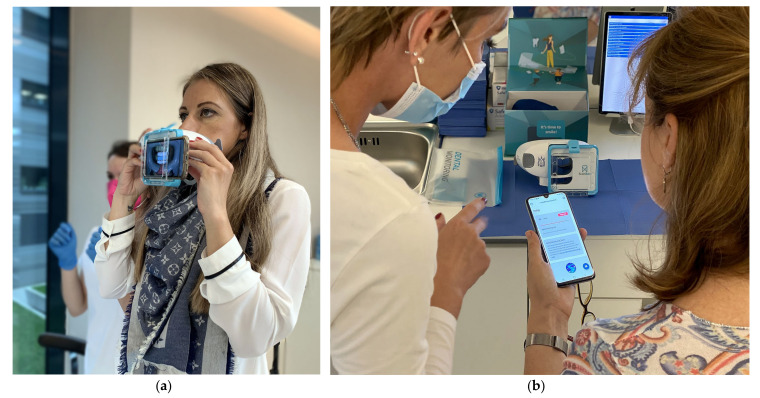
Introduction of Dental Monitoring^®^ (DM): (**a**) Patient holding scan-box looking into the mirror, instructed by nurse, is scanning her first intraoral scan with her own mobile. (**b**) DM has its own app that is used for requests, uploading and reporting of the scans. Its first use is usually also instructed by nurse. The photo was taken for purposes of this article and is published with full written consent of the person.

A typical implementation of TeleHealth systems like Dental Monitoring^®^ (Dental Monitoring Co., Paris, France) require initial patient education. The patients’ own mobile is used for the app and the scanning. First patient downloads the free Dental Monitoring app and activates the free DM app (Figure 1b). Then the first scan is performed with support of nurses in the clinic (Figure 1a). All consecutive video scans are created by patient usually in a home environment. Patient is provided with scan-box that improves quality of video scans (Figure 1a,b).

Dental Monitoring^®^ (Dental Monitoring Co., Paris, France) [39] is described as a software that allows patients to accurately capture their dentition using a patient’s own smartphone and special cheek retractors. A special protocol with GoLive^®^ (Dental Monitoring Co., Paris, France) option in DM is specifically targeted at CAT. Instead of conventional automatic aligner changes, the patients receive a weekly “GO” or “NO-GO” notification. “NO-GO” notification means the clinical situation was not evaluated as suitable for aligner change and patient is expected to stay on the current aligner without further step in the treatment. These notifications are paired with the orthodontists customized pre-recorded instructions. These indicate to patients whether they should proceed to the next aligner or remain in the current one for a few days more. The orthodontist is informed when a NO-GO notification is sent, and individual teeth tracking issues, poor oral hygiene, or broken attachments can be identified. The orthodontist can override a NO-GO if desired. DM has its own app; however, this app is different from the app StrojCHECK^®^ (Bratislava, Slovakia, 3Dent Medical, osim.sk—Society for Medical Innovation (SMI)) analyzed in this research. The DM with its powerful AI capabilities can be used to clinically evaluate the patient clinical performance. Under clinical performance is meant the evaluation of patient’s fit of aligners on teeth. Clinical accuracy of DM is well researched and validated [21,23,24]. 

Use of tele-orthodontics like DM can improve the monitoring of patients during the COVID-19 dissemination [40]. It allowed us to monitor all patients during pandemic lockdowns, reduced the costs and limited direct contact when was not necessary. With all these means it has decreased the risk of COVID-19 dissemination [41].

Attitude of dentists and patients towards the use of Dental Monitoring is positive. Both groups positively judged this tele-orthodontic approach, considering it a technologically advanced tool increasing the perception of quality and accuracy of the treatment [42].

The current state of the research, described above, reviewed aspects of orthodontic health issues, their prevalence and the TeleHealth technologies addressing them. Telemedicine based on orthodontics mobile apps with AI clinical evaluation with Dental Monitoring system is supportive backbone of the future orthodontic care. The next subchapter of introduction describes the researched subject of this research paper—the orthodontic mobile app (strojCHECK) and its AI update that implemented the computerized learning algorithms. And mathematical algorithms, provided by artificial intelligence, continuously boost new therapeutic paradigms [43].

### 1.3. Mobile Application StrojCHECK

Mobile application StrojCHECK^®^ (Bratislava, Slovakia, 3Dent Medical, osim.sk—Society for Medical Innovation (SMI)) is a free smart app for orthodontic patients and doctors. Currently its further development drives the community of medical specialists and other enthusiasts associated under community—Society for Medical Innovation (www.OSIM.sk, accessed on 1 November 2021). The app was originally designed, in 2015 by an orthodontist—MUDr. Andrej Thurzo, PHD, MPH, MHA as simply a solo mobile app without any server background. Its original functions were mostly simple remainders and patient compliance observation. Further it has developed as a solution for complex treatment couching and motivation of orthodontic patients undertaking clear aligner therapy. Application now implements various functions dedicated to support patients on CAT. The app is free to use for everybody, and does not expose users to any form of commercial approach. It requires the clinic/dental office to be registered in the system to manage settings for its patients. 

**Figure 2:**
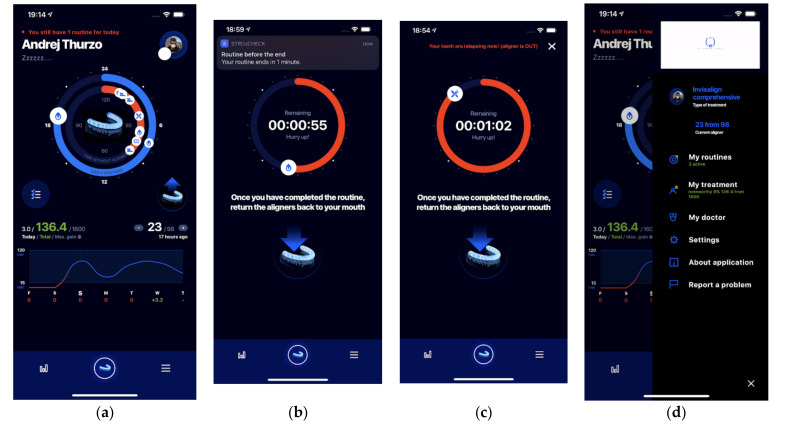
Main screens of orthodontic smart-app StrojCHECK^®^: (**a**) Main dashboard of the app provides complex view of the current day, remaining time, planned and executed activities, lower chart visualizes daily performance of time of aligners out-of-mouth, above it is current balance of earned points/treatment discount and current number of aligner (**b**) Remainders are frequently send as a push notifications to patient mobile and wearable either inquiring if the patient has ended an activity and forgot to return the aligners or is still performing the activity. (**c**) An activity can be finished by active patient interaction (with bonus) or automatically (with sanction) (**d**) Side menu allows user to personalize the app, set up scheduled routines, language, learn about his own performance or contact his doctor directly reporting an event.

In general introduction of this app’s purpose, it can be recapitulated that this smart-app is used for establishing proper treatment routines of orthodontic patients. This app evaluates activities registered by patient, motivates and educates patient enabling him to achieve proper behavioral patterns linked with successful therapy (Figure 1). Central screen is the “Main dashboard” (Figure 2a). This screen provides the complex view of the current day activities, remaining possible time of aligners removed, planned and all executed activities. In the lower chart of this screen there is a visualization of daily performance of time of aligners out-of-mouth. Above this is a current balance of earned points/treatment discount and current number of aligners. Remainder function is frequent feature of many orthodontic apps [10,11]. Remainders are frequently sent as a push notification to patient mobile and wearable either inquiring if the patient has ended an activity and forgot to return the aligners or is still performing the activity (Figure 2b). The activity can be finished by active patient interaction (with bonus) or automatically (with sanction) (Figure 2c). The settings side menu (Figure 2d) allows user to personalize the app and its communication. It provides a management screen for setting up scheduled routines or language. This is the section where patient can learn about his own performance or contact his doctor or nurses directly reporting an event. The events that can be possibly reported include situations of app malfunctions or attachment debonding. Smart functions of the app StrojCHECK^®^ (Bratislava, Slovakia, 3Dent Medical, osim.sk—Society for Medical Innovation (SMI)) and their researched modifications are described in Materials and methods chapter.

### 1.4. Decision Tree Algorithm and Behavior Change Techniques (BCTs)

Smart algorithms that were subject of the app StrojCHECK update and their clinical impact is researched in this paper are introduced in this subchapter.

Mobile apps have been proven to be an effective tool in changing patients’ behavior in orthodontics and can be used to improve their compliance with treatment [44]. In analysis of EU Google play and Apple App Store in August 2021 we have observed approximately 300 orthodontic apps. Some were not fully functional and was difficult to verify their full functionality. In approximately 30 of them the Behavior Change Techniques (BCTs) were observed. This well correlates with the recent findings in publication from September Siddiqui et al. 2021 [44].

The current availability of apps of sufficient quality for patient orthodontic coaching is very limited. There is therefore a need for high-quality orthodontic apps with appropriate BCTs to be created, which may be utilized to improve patients’ compliance with treatment. This paper explores the usefulness and potential of implementation of AI algorithms for enhancing Behavior Change Techniques.

Decision Tree algorithm belongs to the family of supervised learning algorithms. Unlike other supervised learning algorithms, the decision tree algorithm can be used for solving regression and classification problems too.

The goal of using a Decision Tree in general is to create a training model that can be used to predict the class or value of the target variable by learning simple decision rules inferred from prior data (training data).

In Decision Trees, for predicting a class label for a record it begins from the root of the tree. The values of the root attribute are compared to the record’s attribute. On the basis of comparison, the branch corresponding to that value is followed and algorithm jumps to the next node [45]. Types of decision trees are based on the type of target variable available. It can be of two types:

1. Continuous Variable Decision Tree: Decision Tree has a continuous target variable then it is called Continuous Variable Decision Tree.
2. Categorical Variable Decision Tree: Decision Tree which has a categorical target variable then it called a Categorical variable decision tree.

Practical examples of implemented decision tree process algorithms are described in more detail in Materials and methods chapter. 

To keep this introduction comprehensible to scientists outside the field of orthodontic and AI-engineering research, it is necessary to clarify why personalization is currently ideal by means of patient and doctor computerized education. The system allows both groups to improve based on data gathered, evaluated, compared to other users of the system and calculating optimal suggestions. These are set by system autonomously, however from the clinical experience, these might be not optimal for every patient and personalization in this early stage is necessary. 

### 1.5. Aim of This Research Paper

The goal of this research paper is to evaluate AI upgrade of an existing orthodontic mobile coaching app and clinical impact of such upgrade.

Secondary goal is to introduce the advantages of AI (decision tree process algorithm) implementation and method of clinical impact evaluation by means of tele-monitoring systems.

## 2. Materials and Methods

### 2.1. Participants, Statistical Analysis and Hypothesis

86 subjects (54 females and 32 males) in age between 12 and 68 years, were observed with DM 60 days before and 60 days after the AI update of the mobile app they were using. All patients were using the same orthodontic app for treatment coaching—StrojCHECK^®^ (Bratislava, Slovakia, 3Dent Medical, osim.sk—Society for Medical Innovation (SMI)).

The research did not require any approval for human trials as the Dental Monitoring is well clinically established and certified technology [23,26,27,36,37,38] and patients’ mobile application was modified in general (for all patients) with the central update on the official release hubs (App Store for iPhones and Google Play Store for Android mobiles). This was not an interventional study where some of the participants receive different treatment than others in order to evaluate it so there is no control group.

The statistical analysis was performed in Microsoft Office Excel 2016 (Microsoft Corporation, Redmond, WA, USA), Statistica 13.1 software (TIBCO Software Inc., Palo Alto, CA, USA) and StatsDirect 3.3.5 (StatsDirect Ltd., Cheshire, UK).

The collected demographic and clinical, as well as patient-generated data collected through mobile phones were summarized using descriptive statistics. Continuous variables are presented as means with the respective SD (standard deviation) as well as median and interquartile range.

Wilcoxon signed rank test was used to compare the difference in the outcome variables after the upgrading. After-before differences in the outcome variables were computed and regressed to age. The strength of the associations was evaluated by simple bivariate (Spearman’s nonparametric) correlation coefficient.

All presented *p* values were two sided. Values of *p* < 0.05 were considered to indicate a statistically significant difference. Statistical analyses were performed using StatsDirect 3.3.5 (StatsDirect Ltd., Cheshire, UK) statistical software.

The basic hypothesis of this research paper is, that performance of patients interacting with the app (after AI update) will not be significantly different from the time prior to the update.

A possible controversial and diverging hypothesis could be a consideration of a patient using the app only virtually—lying about his true performance. 

This would result in significant discrepancies between the app performance and clinical reality. Despite “lying” to the app is possible, it is extremely frustrating, time consuming and easily detectable.

### 2.2. Description of the Basic Functionality and Workflow Examples

The mobile app StrojCHECK^®^ (Bratislava, Slovakia, 3Dent Medical, osim.sk—Society for Medical Innovation (SMI)) gathers patient data and helps to build a proper routine with focus on the first 120 days of treatment. After 4 months in the treatment, patient usually fixes the routines, even if they are wrong and inappropriate for the treatment. The app communicates with the patients even through their wearables like Apple watch^®^ (Apple Inc., Cupertino, CA, USA), which is very practical. Modern mobile phones include a variety of sensors that can be used to gather data about the user’s behavior [46]. The app functionality is based on these modalities. Mobile app, its back-end and technical background with statistical methods are described in later subchapters. Below are two examples of system intelligent workflows that became possible after the AI-update:

Example 1: Predictions

*Let’s say we have a problem to predict whether a patient will have his CAT interrupted with significant non-tracking of his removable aligners on his teeth (yes/no). Here we know that the “time-without-aligners” of patient is a key—significant variable but the doctor does not have exact time details for all his patients. Now, as we know this is an important variable, then we can build a decision tree to predict patient “time-without-aligners” based on treatment type, occupation, age, app-monitored-interactions, app-monitored-discipline, treatment difficulty and various other variables. In this case, we are predicting values for the continuous variables.*

Example 2: Incentives

*System regularly calculates the most frequent drop-out rate in the use of the app by patients, after 12 days of permanent use, it automatically suggests with push notification to every patient a special motivational badge for completing the continuous streak of daily proper use for another 7 days, however the patient psychology is much more complex and there are various reasons which can be addressed by clinician better who knows the patient personality. So, the doctor is allowed to bypass the system setting and can in particular cases employ a special clinical check-up or another, more suitable form, to bridge a difficult treatment period for the patient. Doctors’ interactions in the system are recorded and will be used later to program separate decision tree algorithm.*

### 2.3. Description of the Supportive Complex System (App StrojCHECK and Its Back-End)

**Figure 3:**
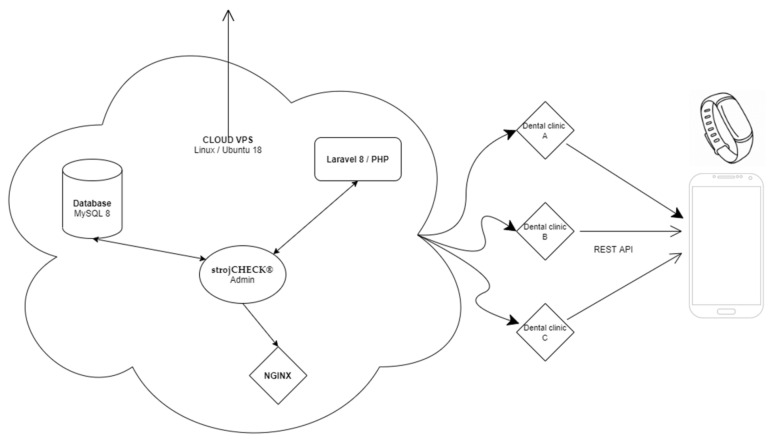
Global schematics of technological platforms fundamental to the complex system of StrojCHECK^®^ from the back-end server through different dental clinics with their separate administrator portals up to end-user devices.

**Figure 4:**
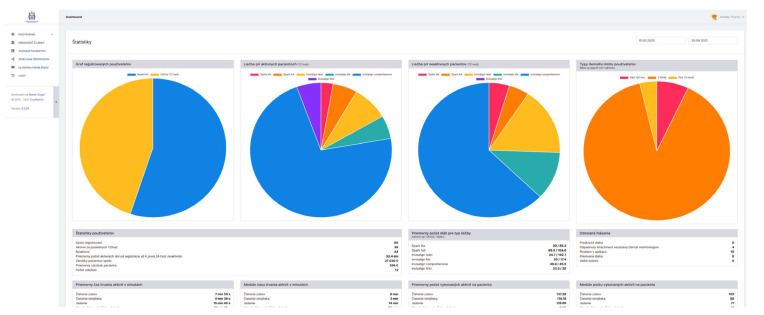
The view of the screen for administration. The portal of the server back-end for doctors and other administrators provides useful interface for statistical data processing and interactions with the system. Pie charts from left describe percentage of active users within the last 72 h (from all registered users), types of monitored appliances in the active users (Spark lite, Spark full, Invisalign teen, Invisalign lite, Invisalign comprehensive, Invisalign first), types of monitored appliances in the non-active users, types of daily limits of removed aligners (over 120 min, within 120 min limit and under 15 min). On the Figure bellow there are visualized user statistics, average times for various habits, Average time per aligner or frequency of patient reports.

The back-end, also called the server side, consists of the server which provides data on request, the application which channels it, and the database which organizes the information (Figure 3). The system consists of three parts:

- The first part is the patient interface in the form of a patient mobile smart application and a possible wearable device.
- The second part of this biomedical system is its “bioinformatic brain”—that is responsible for data gathering, sorting and processing. Also, for some autonomic decision processes and possible future machine-learning algorithms with its own top-admin interface.
- The third part of the system is the admin-interface (Figure 4) for clinical doctors and managers. Here is available the data gathered and processed from mobiles and wearables of their patients. This back-end also allows them to fully customize their clinical set-ups, rules and patient motivation.

The 2nd and 3rd part are running as the software on the server and here is the platform where the future sophisticated AI algorithms will be trained and applied. 

Methods of registration of complex patient behavior on clear aligner therapy depends on way too many variables to be simply programmed. Especially when their statistic evaluation could not be that straightforward. Sophisticated data mining algorithms in the future might be successful in extracting and discovering patterns in large data sets of patients’ behaviors during CAT with other complementary data [47]. 

Methods in apps processing with AI algorithms data images like X-ray, various sounds or ECG have regularly big datasets available that are essential to better training for deeper neural networks [48,49,50,51]. This is not situation for CAT. Variables include not only various behavioral patterns but also different biomechanical approaches on various types of malocclusions. 

### 2.4. Description of the UPDATE (the Set of Computerized Methods Implemented in the Update)

The important part of this research paper is description of the set of computerized methods implemented in the update and later evaluated clinically with Dental Monitoring. All observed patients were using DM, which has its own positive clinical effect [36]. 

StrojCHECK app installed on the mobiles of the users collects tens of thousands of entries every day. This is not only information regarding their eating, drinking or brushing habits. System allows analysis of patterns of patients’ dwindling discipline or their behavioral responses on the motivational events triggered by doctor(admin) or the system itself. We call these events “incentives”.

**Figure 5:**
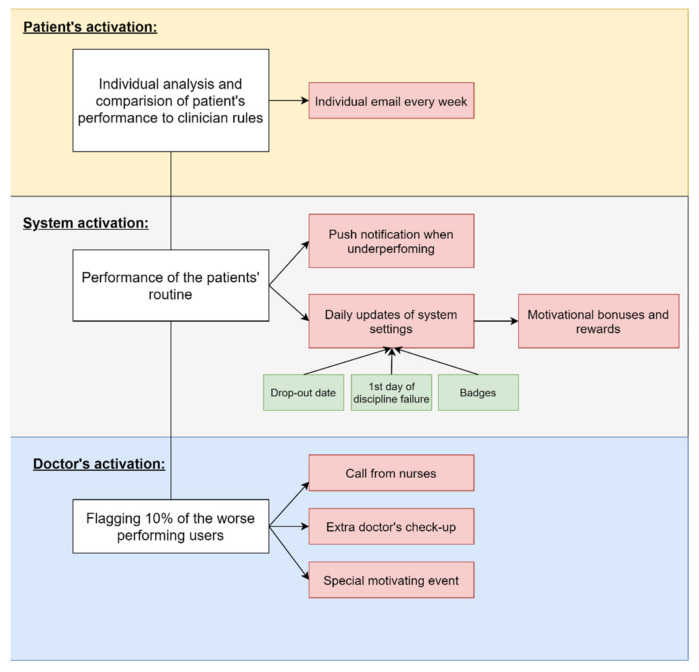
Scheme of three related parts with decision processes that were implemented by the update and represent three layers of system-user interaction.

**Figure 6:**
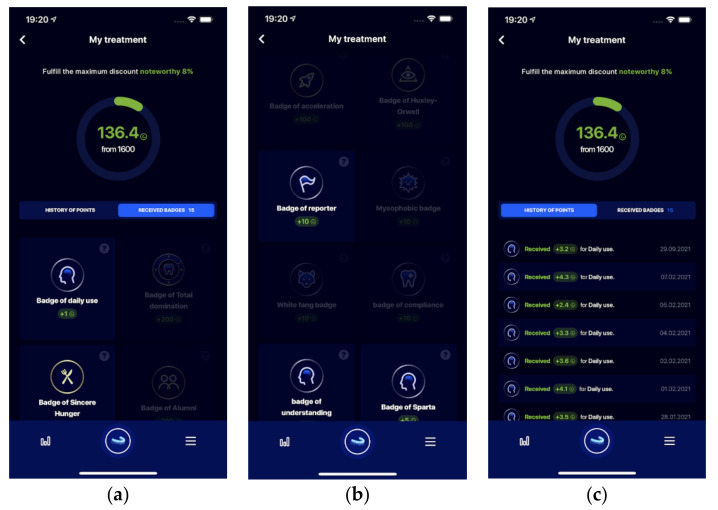
Screens of StrojCHECK^®^ app regarding incentives: (**a**) Patient view of gained points that equal total discount from the treatment budget (**b**) Achieved incentives(badges) and other badges to be achieved are introduced grayed, dynamically introduced by the system. (**c**) List of gained points by following the rules and fulfilling motivational bonuses offered by the system (as special motivational events).

The following three related parts with decision processes were implemented by the update (Figure 5):

(1) **System activation.** System learns when and where the patient does successfully perform her/his routines. When the system notices that the patient is underperforming daily routines or goals the app decides to push the notification in proper time and geo location. These responsivity functions are very simple yet and can be turned off by clinician or the user. System every day after midnight automatically recalculates the settings for various thresholds:
  (a) User drop-out rate (what day most users stop using the app)
  (b) First day of unfulfilled daily rules (what day most users have first discipline failure while using the app)
  (c) Incentives default settings (Badges) (Figure 6.)
    [1] Total domination Badge is achieved after more than X continuous days of proper app use (sum-up).
    [2] Badge of Sincere Hunger achieved after reaching at least X-th day average of more than 5 eating activities per day.
    [3] Badge of Huxley-Orwell, achieved after X-th day of using dental monitoring.
    [4] Badge of Mysophobia, achieved after X days with average aligner cleaning of 3 times per day and more.
    [5] Badge of the White Fang, achieved after X days with average teeth cleaning of 3 times per day and more.
    [6] Badge of compliance, achieved after X-th aligner change and XX days of continuous app use.

System sets the default settings for these stimuli according to recalculated system data. However, these can be changed and fixed by doctor led by his own clinical experience. Otherwise, the system automatically triggers the stimuli—motivational bonuses and rewards to prevent loss of users or their discipline deterioration.

(2) **Doctor activation.** Computerized calculation of various variables, especially time of aligners removed or non-fulfilling the basic criteria (described later) can result in evaluation of patient as underperforming user. 10% of the worse performing users are flagged and clinical team is educated about these patients on a regular basis. Human clinical response to such events most frequently results in extra call from the nurses or extra check-up by the doctor or a special motivational event triggered by doctor manually to prevent the anticipated negative event. The doctor’s behavior in troubleshooting the underperforming patients and the results of such intervention is registered into the system and evaluated. Later the system will learn from it.
(3) **Patient activation.** Computerized analysis of patient performance in relation to all the compulsory clinician rules and also to performance of all other patients using the app was formed as an important tool for patient education. Learning about own weaknesses is first step for improvement. The intelligent system generates and delivers an individualized email report to each patient every 7 days. The report explains the nonperformance events and their probable reasons. Computerized automatic recalculation of patient’s performance can be delivered to patient email and possibly also email of his/her parents. This feature was supposed to help patient learn more about himself/herself and the reason of the failures to prevent them in the future.

Every day, after midnight, the system does calculations and analyses where it evaluates treatments during the previous day. It calculates how many times which patient removed aligners from his mouth, for how long, for what reason, if he returned them automatically within or after the limit, if he needed remainder or how many times the patient broke the rules and hundreds of other calculations for hundreds of patients. System finalizes the calculations by points allocation to each patient according to their performance and compliance with the rules. Points are allocated for each routine activity when interacting with the app (like eating, drinking, cleaning, monitoring etc.). Points can be gained also by special and rare events like computer-game achievements—called badges and also for sharing these badges on their social networks where the patient can boast about special treatment achievements. All previously mentioned points are credited after midnight only if the basic clinical requirements were met. These can be set differently for each participating clinic; however, default rules are required:

▪ clean the teeth at least twice a day (with a gap of 6 h between events)
▪ clean the appliance at least twice a day (with a gap of 6 h)
▪ at least one eating and one drinking
▪ and finally keep all the “aligners-time-out” between 15 and 120 min

### 2.5. Technical Description of the Software Background 

It is built on the latest and greatest available technologies which are popularly used nowadays for mobile app development. Our app architecture is divided into two main departments known as backend and frontend. Backend as the brain of our application, which stores all available data of our patients in the database, calculates and analyses their treatments, and many more logic operations which are necessary for a mobile application to work. Our CRM solution is running in the cloud on Ubuntu/Linux operating system. We use the latest relation database MySQL 8, the programming language is PHP 8 with help of Laravel framework 8, which is popular in the development of a large scale of CRM applications. Our frontend part of the application is running on Android and IOS mobile devices as a mobile application. We developed our mobile application on hybrid mobile app technology, which means we use one shared core of the application in both Ios and Android platforms. In other words, we do not need to develop two separate applications, which reduced the cost and development load of our developers. Our hybrid app is running on HTML5/CSS 3/Javascript and mainly VueJs 3 with the Ionic 5 framework.

## 3. Results

### 3.1. Age and Gender Impact

**Figure 7:**
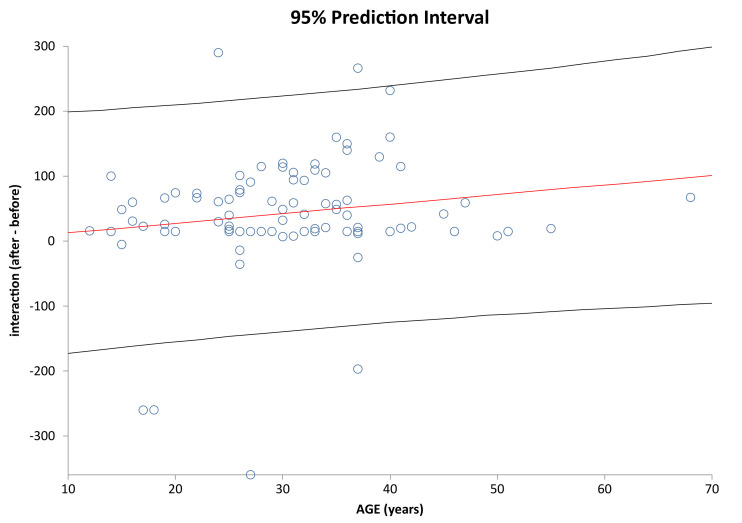
Prediction interval and linear regression line for Interaction (after-before) vs. Age in the investigated sample (correlation coefficient (r) = 0.160; *p* = 0.1416). Each circle represents a data point. The red line is the regression line (the linear model) and the black lines show the 95% prediction band for the forecasted Interaction (after-before).

**Figure 8:**
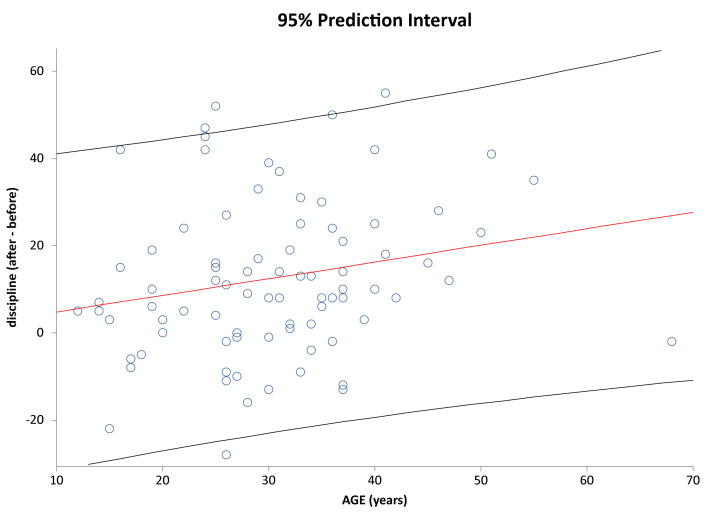
Prediction interval and linear regression line for Discipline (after-before) vs. Age in the investigated sample (r = 0.210; *p* = 0.0528). Regression line and 95% prediction interval are denoted as in Figure 7.

**Figure 9:**
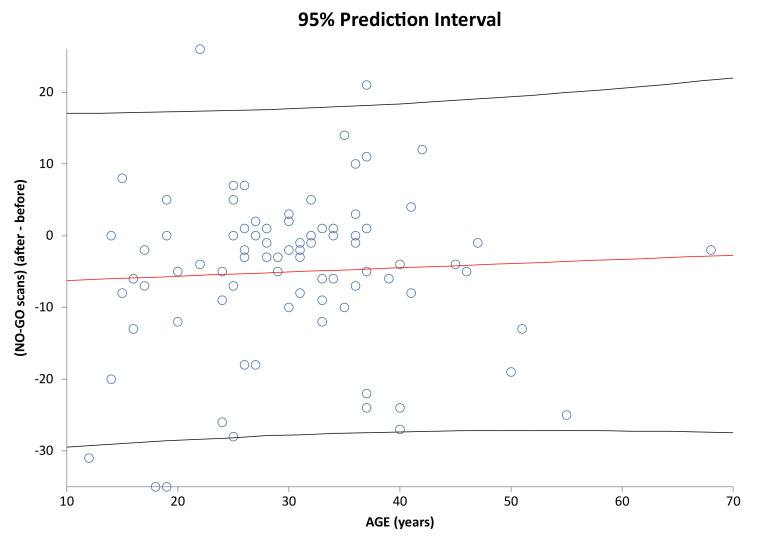
Prediction interval and linear regression line for No-go scans (after-before) vs. Age in the investigated sample (r = 0.0507; *p* = 0.6433). Regression line and 95% prediction interval are denoted as in Figure 7.

**Figure 10:**
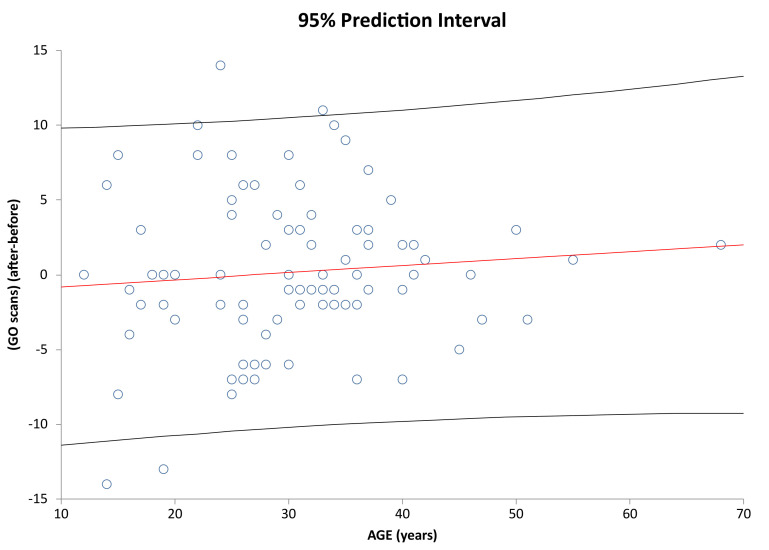
Prediction interval and linear regression line for Age vs. GO scans change (After-Before) vs. Age in the investigated sample (r = 0.0507; *p* = 0.6433). Regression line and 95% prediction interval are denoted as in Figure 7. Coefficient (r) = 0.089831 *p* = 0.4108.

It is analyzed below whether age has an impact on how individual subjects have improved/deteriorated in the given parameters. Figure 7 shows correlation of Age vs. Interaction change after and before the AI update. Figure 8 shows correlation of Age vs. Discipline after and before the AI update. Figure 9 and Figure 10 show correlation of aligner tracking AI evaluation with DM (GO and NO-GO scan evaluations). In short, the results shows that age does not affect the change in any of the monitored parameters.

The influence of gender and differences in individual parameters before vs after and their statistical significance (evaluated by Wilcoxon signed-rank test) is in the Table 2 below. 

### 3.2. Differences of Evaluated Parameters before and after the Update

**Figure 11:**
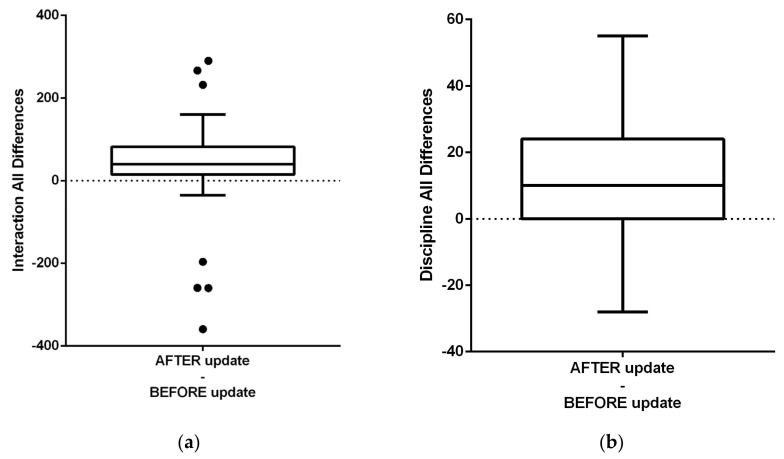
(**a**) Differences before and after update regarding app interaction for all participants. (Paired *t* test of variable Discipline) Measured parameter represented patient app interaction in easy and mostly fun interactions including sharing on social networks or achieving interesting badges. (**b**) Differences before and after update regarding app interaction for all participants (Paired *t* test of variable Discipline). Measured parameter represented patient app interaction in difficult and regular way fulfilling required rules of disciplined use that included teeth and appliance cleaning twice a day (separated by 180 min), at least once per day eating and drinking and as well as fitting the aligner out-of-mouth time between 15 and 120 min. This parameter improved significantly as well.

The graphs in this section present box differences caused by the AI update of the mobile app StrojCHECK^®^ (Bratislava, Slovakia, 3Dent Medical, osim.sk—Society for Medical Innovation (SMI)). Figure 11a shows differences before and after the update in regard to app interaction parameter for all participants. Figure 11b shows differences before and after update regarding app interaction for all participants.

In short, the summary of the results is that there are significant differences in all parameters except GO scans for all and NO-GO scans for boys/men.

**Figure 12:**
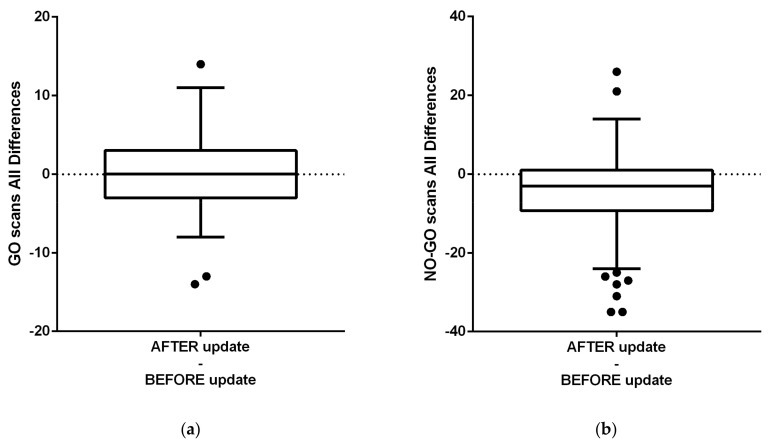
Differences before and after the app update regarding patient clinical performance observed with dental monitoring for all participants. (Paired *t* test) (**a**) Dental monitoring evaluated GO scans focused on proper aligner tracking, here is not a significant difference. (**b**) Dental monitoring evaluated NO-GO scans focused on proper aligner tracking, here is a significant improvement.

**Figure 13:**
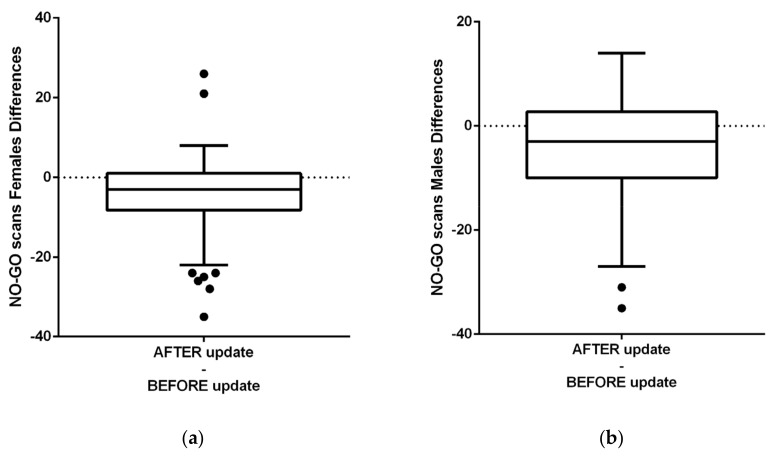
Differences before and after the app update in NO-GO Dental monitoring scans in (**a**) Females (significant) (**b**) Males. Difference, despite were less frequent, is not significant.

The Figure 12a shows differences before and after the app update regarding GO scans for all participants. Figure 12b shows NO-GO scans before and after the update, evaluated with Paired *t* test. Figure 13 shows in (a) females and (b) males’ differences before and after the app AI-update in NO-GO Dental monitoring scans, where in (a) Females (is the difference significant) and in (b) Males is the difference not significant (despite were less frequent).

**Figure 14:**
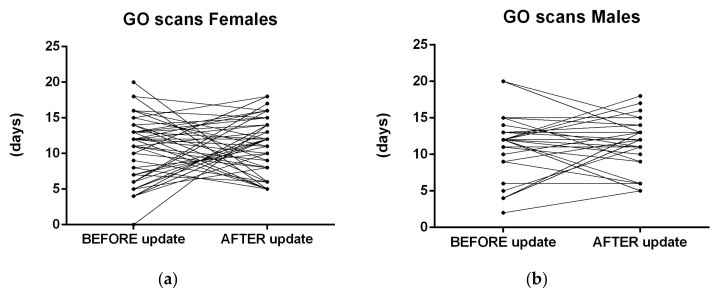
Differences before and after the app update in GO Dental monitoring scans were not significant (**a**) in females (insignificant) (**b**) Males (insignificant).

NO-GO scan is a clinical situation, for this research purposes focused only on “aligner non-tracking” evaluated by AI system of DM. Other reasons of NO-GO scans were not calculated as NO-GO scan, but it was counted into GO-scans group. Figure 14 shows differences before and after the app AI-update in frequency of GO Dental Monitoring scans that were not significant in (Figure 14a) in females (insignificant) and also insignificant in (Figure 14b) Males.

### 3.3. Collateral Interesting Findings

The use of the system and computerized statistical data processing revealed other interesting information, that were not main objectives of this research. To understand the power of such complex TeleHealth app providing support to many patients, is the realization of unprecedented knowledge about the users (patients and doctors). Table 3 shows some of the interesting collateral findings about patient behavior noticed by the system.

## 4. Discussion

This paper focuses on impact evaluation of Computer-based learning (CBL), Decision Tree Algorithms (DTA) and other computerized learning improvements to an existing mobile app—StrojCHECK^®^ (Bratislava, Slovakia, 3Dent Medical, osim.sk—Society for Medical Innovation (SMI)). 

The main aim of the research was to evaluate clinical impact of implementation of decision processes to an existing healthcare application for orthodontic treatment. The secondary goal was to use AI system (DM) to evaluate the clinical situation in high frequency established on video scans of patient teeth and their appliances. 

The results invalidated the basic hypothesis of this research paper. That performance of patients interacting with the app (after the AI update) will not be significantly different from the time prior to the update.

With the use of decision processes methods applied in an existing healthcare app, unprecedented data about orthodontic patients’ behavior are available for analysis. New technique for clinical research using patients’ made video-scans has been presented as successful scientific tool [37]. The information about application StrojCHECK was never published before. It represents a complex healthcare system driven by computational methods. 

Results show that impact of the app is significant in evaluation of patient—app interaction. The amount of GO scans is not significantly reduced and this might be explained due to the fact, that frequency of scans is defined by doctor and if everything goes well, the patient will go as prescribed and do the scan regularly. 

The decrease of frequency of NO-GO scans is slightly significant only in women and not in man. This can be interpreted by fact that farther the patient goes into the treatment there is higher probability of discrepancy between teeth positions and aligners. So simply the clinical development shall deteriorate over time.

Results also confirmed that change in performance was significant in all observed parameters except the GO scans and NO-GO scans for males. Results also showed they are unrelated to the age. In general, the AI update of the app resulted significantly better clinical performance. The clinical variables like insignificant change in GO scans can be explained that first 60 days of treatment contains usually the better compliance and as well as the tracking of the patient that worsens in time. The clinical non-tracking gets worse over time naturally. It is the fact that GO scans frequency is defined by rigid doctor instruction and shall not change unless doctor indicates to speed up the protocol. 

Result in insignificant improvement of NO-GO scans in males is showing the discrepancy of their increased app-activity regarding interactions with the app as well as the discipline, however without clinical effect. This might be explained by natural lag of clinical performance as well as rule described above regarding worsening clinical performance of tracking over-time.

In context to other publications, it is known the App update on the verge of AI can have a significant effect on its user behavior [28,44]. The success of further app development relies on understanding of the patient (user) behavior. CBL is a term used for any kind of learning with the help of computers. Computer-based learning makes the use of the interactive elements of the computer applications and software and the ability to present any type of information to the users. Authors of this paper have published their experience in smart learning algorithms implementation [52,53], albeit any sophisticated A.I. upgrade requires simple computerized learning features first. 

TeleHealth solutions are currently driven with AI implementations in various medical fields from treatments of chronic pulmonary diseases, depressions or intellectual disabilities to other chronic diseases [54,55,56,57,58,59,60,61].

Another highlight of this study is that not necessarily a universal approach to modification of patient behavior would result in better clinical results as every patient behavior is unique and we need to differentiate between behavioral patterns of our patients first, program the proper motivational responses later. So, the concept was to invest the benefits of first intelligent features of the system into doctor module and patient module of the app. Under this we understand to provide the data of particular patient behavior to the doctor and allow him to adjust the motivational responses of the system as well as provide regularly the information about the patient’s weaknesses to the patient himself. 

Dentistry needs to expand its understanding of how dental apps; digital workflow models and digital health information are transforming dental practice in order to anticipate how this digital shift will impact the whole field of dentistry [62].

As has been stressed in this article repetitively—the CAT success strongly depends not only on patient motivation and discipline but also on patient’s understanding of the basic treatment rules and his own weaknesses. These are frequently unknown to patient and often also to his doctor. 

Recently published retrospective cohort study in the Journal of Clinical medicine presented interesting findings about factors influencing patient compliance during CAT [63]. These also can be used to guide practitioners towards limitedly compliant individuals, allowing early intervention and later to help program a sophisticated A.I. algorithms.

Clear aligners weakness is patient forgetfulness. As the CAT’s effectivity is compromised when aligners are not on patient’s’ teeth for longer than 120 min per day. Patient removes them for cleaning the teeth and appliance, or during eating and drinking especially coloring food and drinks [64].

In wider context, authors of this paper were searching for a complex personalized solution that would significantly improve patient treatment compliance by the means of using a smart healthcare app. Today it seems impossible to achieve this without implementation of smart computerized data processing algorithms.

The big data automatically collected by the system is crucial for future machine learning. Analysis of the daily routines of CAT patients and understanding the patterns of their discipline, motivation, bad habits and behavioral responses to stimulus are foundation to successful future programming of more advanced AI algorithms. The goals of future AI implementation shall be:

1. Early identification of non-compliant patient
2. Anticipation of incoming drop of discipline according to app use pattern
3. Designing ideal treatment patterns for specific patient types upon their behavioral as well as clinical parameters
4. Designing ideal motivational impulses according to patient type, daily routines and specifics of his/her particular treatment

## 5. Conclusions

The principal conclusion of this research is that implementation of AI decision processes algorithm is not only the first step towards more sophisticated machine-learning decision optimization models, but also an already effective enhancement of an existing app with measurable benefits to patients. This conclusion is based on the results of this research as in all pairs of monitored variables was observed a significant improvement except for AI evaluation of clinical tracking of aligners in men.

Secondary conclusion is that Dental Monitoring is a useful tool in evaluation of clinical situation on the principles of telemedicine.

## Figures and Tables

**Table 1:** The characteristics of current studies about apps used in orthodontics (*n* = 20).

Study Type	Author	Domain of Use	Focus Group of Apps
RCT	Alkadhi [9] 2017	Reminders	Patient
RCT	Deleuse [10] 2020	Reminders	Patient
RCT	Li [11] 2016	Reminders	Patient
RCT	Scheerman [12] 2020	Reminders	Patient
RCT	Zotti [13] 2016	Reminders; remote monitoring	Patient; clinician
RCT	Zotti [14] 2019	Reminders; remote monitoring	Patient; clinician
RCT	Al-Abdallah [15] 2021	Reminders	Patient; clinician
RCT	Ross [16] 2019	Reminders	Patient
Case-control	Abdul Khader [17] 2020	Diagnostics	Clinician
Case-control	Aksakalli [18] 2017	Diagnostics	Clinician
Case-control	Goracci [19] 2014	Diagnostics	Clinician
Case-control	Kumar [20] 2020	Diagnostics	Clinician
Case-control	Kuriakose [21] 2019	Remote monitoring	Patient; clinician
Case-control	Livas [22] 2019	Diagnostics	Clinician
Case-control	Morris [23] 2019	Remote monitoring	Patient; clinician
Case-control	Moylan [24] 2019	Remote monitoring	Patient; clinician
Case-control	Sayar [25] 2017	Diagnostics	Clinician
Case-control	Caruso [26] 2021	Remote monitoring	Patient; clinician
Retrospective cohort study	Hansa [27] 2020	Remote monitoring	Patient; clinician
Cross-sectional	Underwood [28] 2015	Reminders	Patient

**Table 2:** The influence of gender and differences in individual parameters before vs after the AI update and their statistical significance (evaluated by Wilcoxon signed-rank test).

Parameter	Statistics	Females			Males			All		
		Before	After	Difference (After-Before)	Before	After	Difference (After-Before)	Before	After	Difference (After-Before)
Interaction	*n*	54	54	54	32	32	32	86	86	86
	Mean	166.7	204.8	38.07	99.71	151.5	51.78	141.8	185	43.17
	Std. Deviation	190.3	153.3	110	99.85	128.1	44.46	165.2	146	91.18
	Minimum	18	13.25	−359.3	15	35.75	8.25	15	13.3	−359.3
	25% Percentile	50	83.19	15	50	65	15	50	65	15
	Median	78.7	149.3	40.06	50	97.25	37.17	64.63	141	40.06
	75% Percentile	223.5	293.8	93.85	101.6	164.8	75.9	197.2	275	82
	Maximum	802.4	544.4	290.2	432.6	562.3	160.2	802.4	562	290.2
	*p*-value			<0.0001			<0.0001			<0.0001
Discipline	Mean	34.7	47.44	12.74	35.22	47.59	12.38	34.9	47.5	12.6
	Std. Deviation	19.44	16.57	19.7	18.46	17.83	15.07	18.97	17	18.02
	Minimum	0	2	−28	1	0	−16	0	0	−28
	25% Percentile	17.5	45.75	−2	19.25	42	3.5	18.75	45.8	0
	Median	34.5	55	11	37.5	56	9	36	55	10
	75% Percentile	55	59	25.5	51.75	59	18.75	53.25	59	24
	Maximum	60	60	55	60	60	50	60	60	55
	*p*-value			<0.0001			<0.0001			<0.0001
NO-GO scans	Mean	11.43	6.167	−5.259	11.44	6.813	−4.625	11.43	6.41	−5.023
	Std. Deviation	10.82	6.911	11.24	11.53	6.64	11.6	11.02	6.78	11.31
	Minimum	1	0	−35	1	0	−35	1	0	−35
	25% Percentile	3	1	−8.25	3.5	1.25	−10	3	1	−9.25
	Median	7	4	−3	6	5	−3	7	4	−3
	75% Percentile	19.75	11	1	15	12	2.75	15.25	12	1
	Maximum	45	30	26	50	25	14	50	30	26
	*p*-value			0.0002			0.0525			<0.0001
GO scans	Mean	10.87	11.15	0.2778	11.44	11.41	−0.03125	11.08	11.2	0.1628
	Std. Deviation	4.112	3.779	5.738	3.975	3.301	4.092	4.047	3.59	5.163
	Minimum	0	5	−14	2	5	−7	0	5	−14
	25% Percentile	7.75	8	−3	10.25	10	−2	9	9	−3
	Median	12	12	0	12	12	0	12	12	0
	75% Percentile	13	14	3.25	13	13	3	13	14	3
	Maximum	20	18	14	20	18	9	20	18	14
	*p*-value			0.7029			0.9207			0.7692

**Table 3:** Information from statistical data processing of the large set of patients.

Activity ^1^	Median Session Length	Average Time
Teeth cleaning	6 min	8 min
Eating	14 min	
Drinking	6 min	
Appliance cleaning 2	3 min	
Dental monitoring	5 min	

^1^ Activity describes only one uninterrupted session.

## Data Availability

Data available in a publicly accessible repository that does not issue DOIs. Publicly available datasets were analyzed in this study. This data can be found here on the following link: https://bit.ly/3pPJCEr (accessed on 6 December 2021).

## Abbreviations

app	Mobile application
AI	Artificial Intelligence (A.I.)
BCT	Behavior Change Techniques (BCTs)
CBL	Computer-based learning
CAT	Clear Aligner Therapy
DTA	Decision Tree Algorithms
DM	Dental Monitoring
RCT	Randomized Controlled Trial
